# Role of Calcitonin Gene-Related Peptide in Functional Adaptation of the Skeleton

**DOI:** 10.1371/journal.pone.0113959

**Published:** 2014-12-23

**Authors:** Susannah J. Sample, Caitlin M. Heaton, Mary Behan, Jason A. Bleedorn, Molly A. Racette, Zhengling Hao, Peter Muir

**Affiliations:** 1 Comparative Orthopaedic Research Laboratory, School of Veterinary Medicine, University of Wisconsin-Madison, Madison, Wisconsin, United States of America; 2 Department of Comparative Biosciences, School of Veterinary Medicine, University of Wisconsin-Madison, Madison, Wisconsin, United States of America; University of Notre Dame, United States of America

## Abstract

Peptidergic sensory nerve fibers innervating bone and periosteum are rich in calcitonin gene-related peptide (CGRP), an osteoanabolic neurotransmitter. There are two CGRP isoforms, CGRPα and CGRPβ. Sensory fibers are a potential means by which the nervous system may detect and respond to loading events within the skeleton. However, the functional role of the nervous system in the response of bone to mechanical loading is unclear. We used the ulna end-loading model to induce an adaptive modeling response in CGRPα and CGRPβ knockout mouse lines and their respective wildtype controls. For each knockout mouse line, groups of mice were treated with cyclic loading or sham-loading of the right ulna. A third group of mice received brachial plexus anesthesia (BPA) of the loaded limb before mechanical loading. Fluorochrome labels were administered at the time of loading and 7 days later. Ten days after loading, bone responses were quantified morphometrically. We hypothesized that CGRP signaling is required for normal mechanosensing and associated load-induced bone formation. We found that mechanically-induced activation of periosteal mineralizing surface in mice and associated blocking with BPA were eliminated by knockout of CGRPα signaling. This effect was not evident in CGRPβ knockout mice. We also found that mineral apposition responses to mechanical loading and associated BPA blocking were retained with CGRPα deletion. We conclude that activation of periosteal mineralizing surfaces in response to mechanical loading of bone is CGRPα-dependent *in* *vivo*. This suggests that release of CGRP from sensory peptidergic fibers in periosteum and bone has a functional role in load-induced bone formation.

## Introduction

Fractures associated with development of osteoporosis or bone fatigue from microdamage accumulation are an economically important healthcare problem in the United States [1]. Pathological fractures develop in adapted bone when application of cyclic strains lower than those required to fracture normal bone in a single cycle lead to fracture [2]. Stress fractures are common in human athletes, army recruits, and cursorial animals [3]–[5], and cause considerable expense for the military [6]. The skeleton is exquisitely sensitive to loading and functional adaptation occurs in response to minimal cyclic load and strain [7]. However, physiological signaling pathways with mechanosensory function are not clearly defined in bone. The specific reason(s) why functional adaptation often fails to protect the skeleton from injury are not fully understood.

It has been widely concluded that the osteocyte is the primary mechanosensory cell in bone. Detection of mechanical strain by osteocytes fits well with the view that skeletal adaptation is a local phenomenon. Osteocyte physiological responses to load or the absence of load [8], to microdamage [9], and to interstitial fluid flow [10] may be important in this regard.

Afferent sensory nerve fibers are also a potential means by which the nervous system may detect loading events within the skeleton. In the past, nerve endings in bone have not been considered important for bone mechanosensing [11]. However, the periosteum, and to a lesser degree, cortical bone, is innervated with a dense meshwork of nerve fibers optimized for detection of mechanical distortion [12], [13]. These tissues contain sensory nerves that release a range of neuropeptides and neurotransmitters, including calcitonin gene-related peptide (CGRP) [14]. There are two isoforms of CGRP, CGRPα and CGRPβ [15]. In-vitro experiments suggest that CGRPβ, unlike CGRPα, does not have osteogenic effects [16].

Previous research from our laboratory suggests that mechanosensing responses during functional adaptation of appendicular bone to mechanical loading are not exclusive to osteocytes, and also involve the nervous system [17], [18]. Unmyelinated sensory nerves establish direct connections between individual bone cells and the brain [19], potentially enabling direct neural regulation of bone physiology. Peptidergic neurotransmitters are enriched in sensory fibers in bone [20]. Site-specific sprouting of peptidergic sensory fibers is associated with fracture healing responses in bone. In an angulated fracture healing model, bony callus forms preferentially on the compressive concave bone surface in association with site-specific sprouting of CGRP-positive (CGRP^+^) nerve fibers [21]. During fracture remodeling, the density of substance P-positive and neuropeptide Y-positive fibers is related to bone resorption [22], [23].

Although it is generally accepted that neuronal signaling has regulatory effects on bone metabolism [24], the functional role of the nervous system on the response of bone to mechanical loading is unclear. In a brachial plexus anesthesia rat model, temporary blockade of neuronal signaling induces inhibition of load-induced bone formation in response to mechanical loading of the ulna [17]. Furthermore, bone CGRP concentrations are increased in response to cyclic ulna loading [25]. Collectively, these observations suggest that neuronal signaling regulates functional adaptation to mechanical loading, and suggests that peptidergic sensory nerves, particularly CGRP^+^ fibers, are important in this regard. The present study was designed to determine whether CGRP signaling is required for normal mechanosensing within bone and associated functionally adaptive bone formation.

## Materials and Methods

### Animals

Groups of male CGRPα and CGRPβ knockout and wildtype mice aged 19–21 weeks of age were used for the study. Mice were provided with food and water ad libitum. Mice were housed in a purpose-designed temperature and humidity controlled facility with a 12-hour light/dark cycle. Bedding material and a plastic house or a tube was placed in the cage for environmental enrichment. Daily examinations were performed on all animals throughout the experimental period. CGRPα knockout and wildtype mice were bred on a C57BL/6 background. CGRPβ knockout and wildtype mice were bred on a Swiss background. 193 mice were used for the experiments.

### Ethics statement

All procedures were performed in strict accordance with the recommendations in the Guide for the Care and Use of Laboratory Animals of the National Institutes of Health and the American Veterinary Medical Association and with approval from the Animal Care Committee of the University of Wisconsin-Madison (V1148).

### Experimental design

Groups of knockout (n = 45) and wildtype mice (n = 51) with a C57BL/6 background were used for the studies of CGRPα. Groups of knockout (n = 56) and wildtype mice (n = 61) with a Swiss background were used for studies of CGRPβ. A subset of mice within each group was used as a sham control. All other mice had their right ulna loaded. Mice were placed into groups via block randomization. A proportion of the loaded mice in each group were given perineural anesthesia of the right brachial plexus with bupivacaine (BPA) before loading, to induce temporary neuronal blocking between the limb and the spinal cord. All mice received a subcutaneous injection of calcein green (20 mg/kg) at the time of loading, and an intra-peritoneal injection of alizarin red (40 mg/kg) 7 days later. Humane euthanasia of mice was performed under isoflurane anesthesia using intracardiac injection of pentobarbitone (150 mg/kg) at the end of the 10-day experimental period. Humane euthanasia was also performed in 23 of 30 mice excluded from the study analyses. Of the remaining 7 animals, 4 mice died under general anesthesia without receiving intracardiac pentobarbitone injection and 3 mice were found dead in their cage during the study.

### *In-vivo* ulnar loading

**Figure 1:**
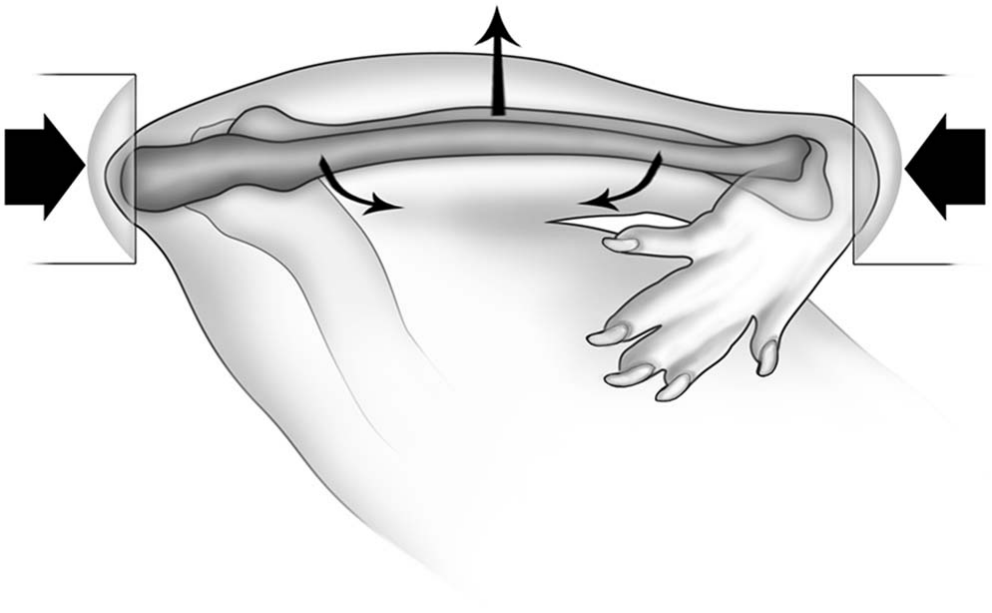
Schematic diagram of the ulna loading model. The antebrachium was placed horizontally in loading cups attached to a materials testing machine. The medio-lateral diaphyseal curvature of the ulna is accentuated through axial compression, most of which is translated into a bending moment. Reproduced from [18] with permission from John Wiley & Sons.

*In-vivo* loading of the right ulna was performed under isoflurane-induced general anesthesia. Butorphanol analgesia (0.5 mg/kg) was given before anesthetic induction and again before recovery from general anesthesia. The right antebrachium of each mouse was placed horizontally between two loading cups, which were fixed to the loading platen and actuator of a materials testing machine (Model 8800 DynaMight; Instron, Canton, MA, USA) with a 10 N load cell (Honeywell Sensotec, Canton, MA, USA). The right ulna then underwent cyclic loading by means of axial compression, which accentuates the pre-existing mediolateral curvature of the diaphysis of the mouse ulna, translating most of the axial force into a bending moment (Fig. 1). To determine the relationship between peak load and initial peak strain for the C57BL/6 and Swiss mice in this model, we performed an *ex-vivo* study using 4–5 mice of each wildtype and knockout line. A single rosette strain gage (EA-06-031DE-120, 120 Ω; Vishay Micromeasurements, Malvern, PA, USA) was bonded to the diaphysis of the caudal medial surface of the right ulna at 60% of bone length from the proximal end of the bone. The right ulna was cyclically end-loaded in compression at 2 Hz for a small number of cycles (50 cycles at each load) using a series of compressive loads [17]. All mice were loaded for 800 cycles using a 2 Hz haversine wave. As a result of these strain gage data, the following loads were used: wildtype C57BL/6 mice were loaded at −2.54 N, knockout C57BL/6 mice were loaded at −2.49 N, wildtype Swiss mice were loaded at −2.12 N and knockout Swiss mice were loaded at −2.0 N. These loading regimens resulted in approximately −3,500 µe at 60% total bone length measured from the proximal end of the ulna and induced lamellar bone formation.

### Brachial Plexus Anesthesia

Perineural anesthesia of the nerves of the right brachial plexus was performed 5 min before loading using bupivicaine (Marcaine 0.5%; Hospira, Lake Forest, IL, USA) [17] at a dose of 6.6 mg/kg. A train-of-four nerve stimulator (Micro Stim; Neuro Technology, Houston, TX, USA) was used to confirm correct positioning of the insulated injection needle (ProBloc II; Portex, Smiths Medical, St Paul, MN, USA). Functional blocking of neuronal signaling between the spinal cord and the loaded limb was confirmed by observing temporary paralysis of the limb on recovery from anesthesia, which resolved within 1 hour of loading.

### Bone histomorphometry

After euthanasia, pairs of ulnae were dissected along with surrounding tissue. Bones were dehydrated in ethanol (70% and then 100%), and embedded in methylmethacrylate. Ulnae were sectioned at 60% of total bone length measured from the proximal end. Transverse calcified sections, 125 µm thick, were made and mounted on standard microscope slides. Confocal microscopy (MRC-1024 Laser Scanning Confocal Microscope; Bio-Rad, Hercules, CA, USA) was used to collect fluorescent images of each bone section. Classical morphometric analysis was used (Image J; NIH), including periosteal and endosteal mineralizing surface (MS/BS, %), mineral apposition rate (MAR, µm/day), and bone formation rate (BFR/BS, µm^3^/µm^2^/yr). All periosteal (Ps) measurements were made by a one observer (SJS) and all endosteal (En) measurements were made by a second observer (CMH). Relative bone formation was calculated by subtracting the left contralateral value from the equivalent right value from the loaded limb (rMS/BS, rMAR, rBFR/BS).

### Dual-energy x-ray absorptiometry scanning

Whole body dual-energy x-ray absorptiometry scanning was performed using a PIXImus densitometer (GE Lunar, Madison, WI). Groups of CGRPα and CGRPβ knockout and associated wildtype mice were scanned (n = 5) and body composition was determined.

### Statistical analysis

Data are reported as mean ± standard error of the mean. The Kolmogorov-Smirnov test was used to confirm that data were normally distributed. Right and left limbs were treated as separate experiments. In each mouse strain, a two-way ANOVA with a Fisher’s LSD post-hoc test was used to determine treatment effects in loaded and contralateral bones and relative right-left differences for each loading treatment (Sham, Load, or Block+Load). Right-left comparisons were considered repeated-measures in these analyses. Differences in body composition between mouse strain were also analyzed with a two-way repeated measures ANOVA with a Fisher’s LSD post-hoc test. The Student’s *t* test was used to determine differences in bone formation between wildtype and CGRPα and CGRPβ knockout mouse ulnae for each treatment group and limb. Results were considered significant at *p*<0.05.

## Results

No evidence of fatigue damage, including woven bone formation or the presence of microdamage, was found in any bone sections upon microscopic evaluation. A total of 30 mice were excluded from histopathologic analysis of bone formation for the following reasons: incomplete BPA, as determined by the animals having motor function of the right thoracic limb upon recovery from general anesthesia (15), lack of fluorochrome uptake (4), death under anesthesia (4), death in cage due to suspected or necropsy confirmed septic peritonitis (3), premature euthanasia due to wounding by a cage-mate (2), and loading error (2).

### Load-induced bone formation responses are different in CGRPα wildtype and knockout mice

**Figure 2:**
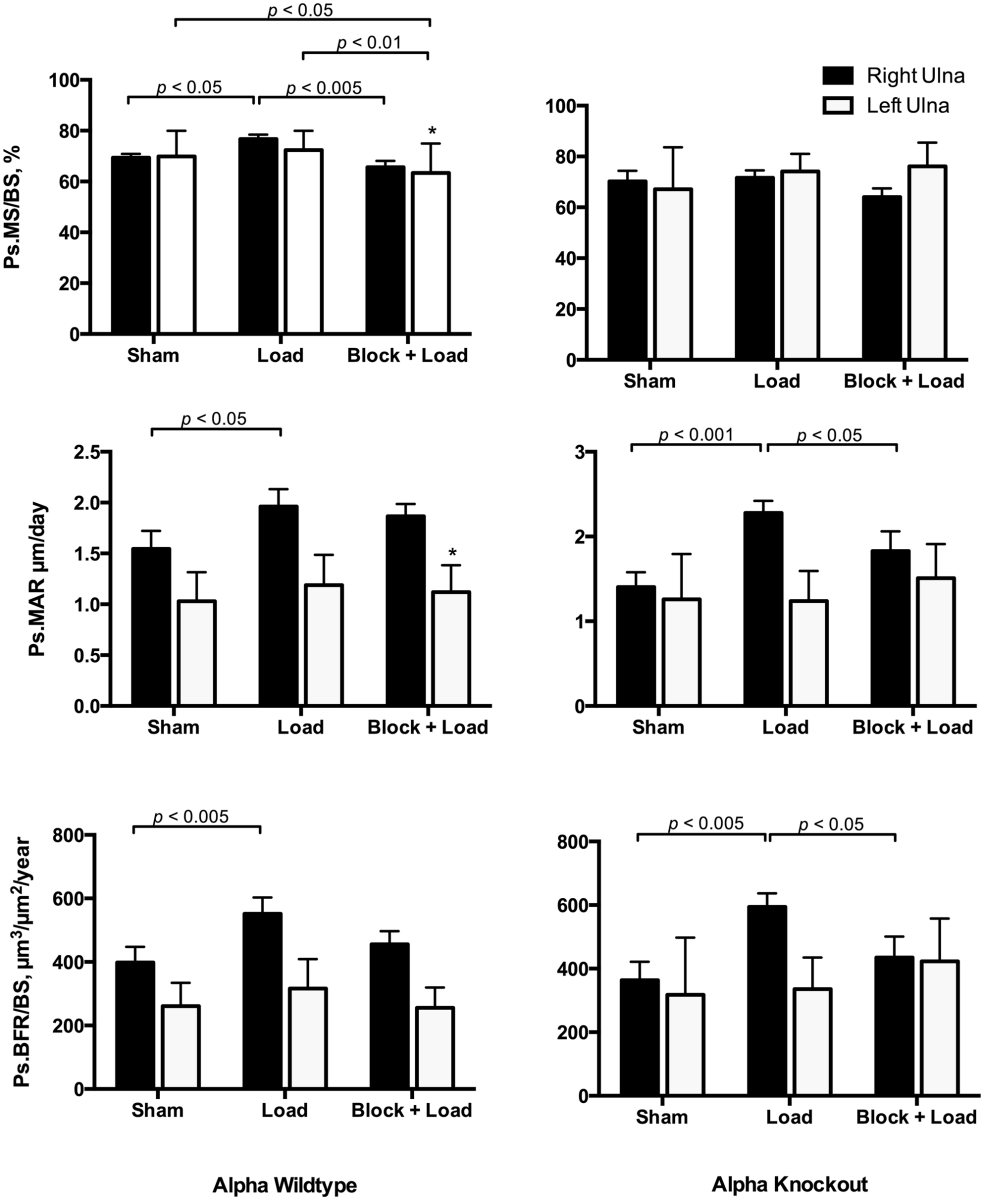
Load-induced periosteal bone formation responses are different in CGRPα wildtype and knockout mice. Cyclic loading of the right ulna in wildtype mice induced adaptive responses in periosteal mineralizing surface (Ps.MS/BS), periosteal mineral apposition rate (Ps.MAR), and periosteal bone formation rate (Ps.BFR/BS). When brachial plexus anesthesia (BPA) was performed before loading, the Ps.MS/BS response to loading was significantly blocked (*p*<0.005). In CGRPα knockout mice, Ps.MS/BS response to loading was lost, but Ps.MAR and Ps.BFR/BS response to loading was preserved. Blocking of Ps.MAR and Ps.BFR/BS responses in the right ulna after right ulna loading was enhanced in CGRPα knockout mice, compared with wild-type mice. *p<0.05 versus left ulna in Block + Load CGRPα knockout mice. Sham – sham loaded group, Load – loaded group, Block + Load – BPA treatment before loading. n = 11–14 mice/group.

**Figure 3:**
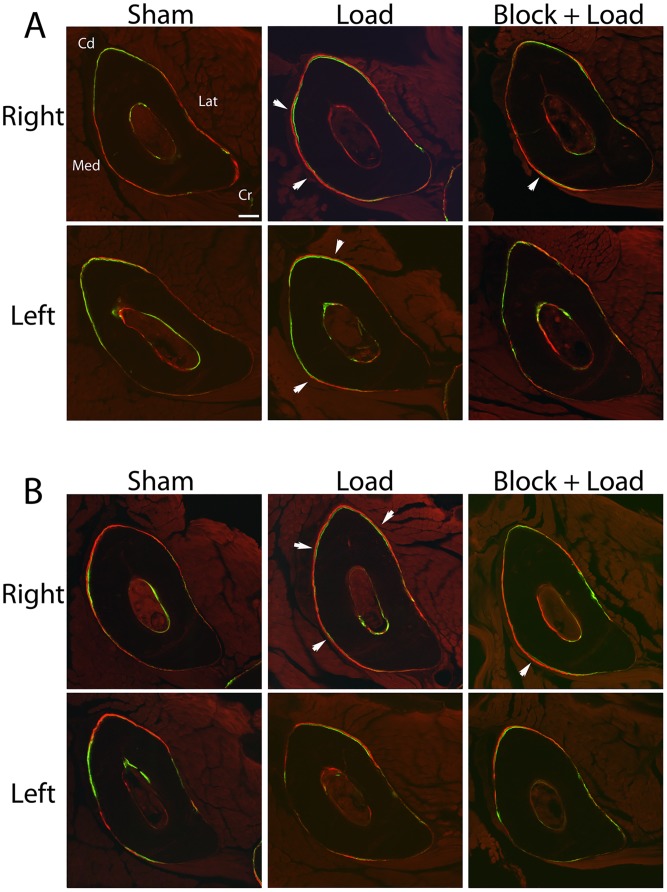
Fluorochome-labeled new bone formation in CGRPα wildtype and knockout mice. Cyclic loading of the right ulna in wildtype (**A**) and knockout mice (**B**) induced an adaptive response with increased periosteal bone formation (white arrows). Blocking of periosteal bone formation with responses was more evident in the CGRP**α** knockout mice. Bar = 110 µm. Cd, caudal; Cr, cranial; Lat, lateral; Med, medial. Sham – sham loaded group, Load – loaded group, Block + Load – BPA treatment before loading. n = 11–14 mice/group.

**Figure 4:**
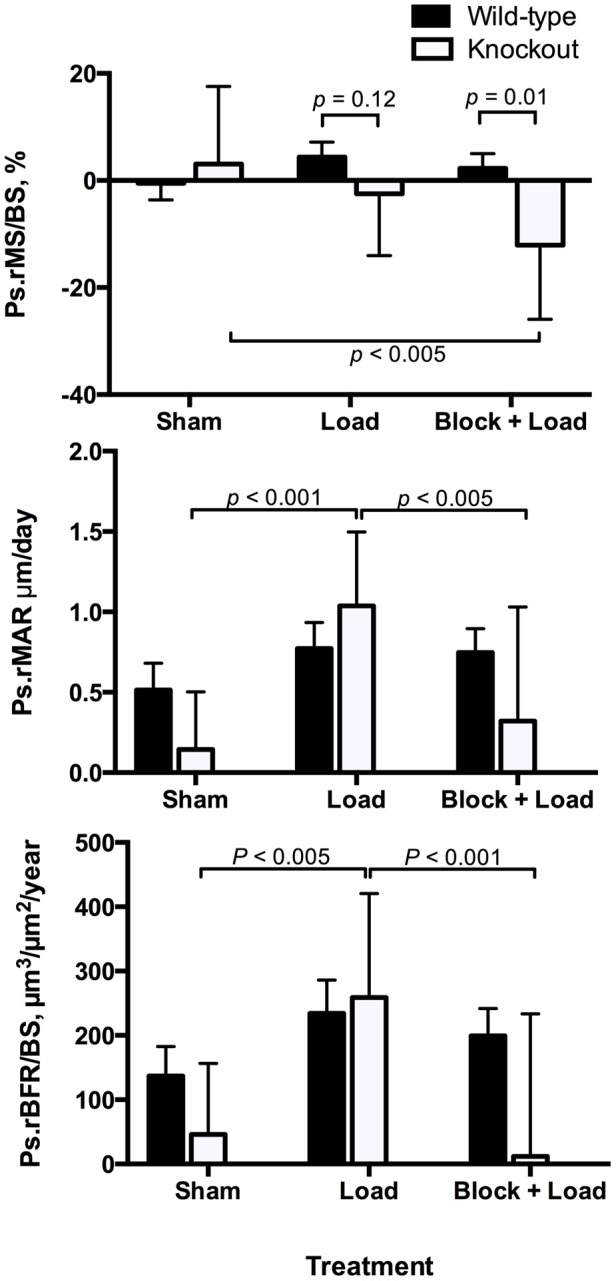
Adaptive periosteal bone responses in CGRPα wildtype and knockout mice are mainly influenced by changes in mineral apposition rate. Cyclic loading of the right ulna in CGRPα knockout mice, but not wild-type mice, induced significant changes in relative periosteal mineral apposition rate (Ps.rMAR). Blocking of periosteal relative mineral apposition rate (Ps.rMAR) and periosteal relative bone formation rate (Ps.rBFR/BS) by brachial plexus anesthesia (BPA) was also found in CGRPα knockout mice, but not wildtype mice. Sham – sham loaded group, Load – loaded group, Block + Load – BPA treatment before loading. n = 11–14 mice/group.

In CGRPα wildtype mice, ulnar Ps.MS/BS, Ps.MAR, and Ps.BFR/BS were increased in the loaded right ulna in response to loading (*p*<0.05); the Ps.MS/BS response was blocked with BPA (*p*<0.005) (Figs. 2 and 3). In the contralateral ulna, Ps.MS/BS, but not Ps.MAR or Ps.BFR/BS was also decreased after BPA in CGRPα wildtype mice, when compared to the Sham and Load groups (*p*<0.05). Ps.rMS/BS was significantly suppressed in CGRPα knockout mice in response to BPA, compared to the Sham group (*p*<0.005) (Fig. 4). The increase in Ps.rMAR and Ps.rBFR/BS with bone loading was significant in CGRPα knockout mice (*p*<0.005), but not wildtype mice (Fig. 4). BPA resulted in a significant decrease in Ps.rMAR and Ps.rBFR/BS in the knockout mice (*p*<0.005) but not the wildtype mice, when compared to their respective Load groups (Fig. 4). In addition, Ps.MS/BS in the left ulna of CGRPα wildtype mice after BPA was decreased relative to CGRPα knockout mice (*p*<0.05) (Fig. 2). In the loaded right ulna of CGRPα knockout mice, Ps.MAR and Ps.BFR/BS were decreased in the Block+Load group, when compared with the Load group (*p*<0.05) (Fig. 2). The relative contribution of Ps.MS/BS to periosteal bone formation was higher in wildtype CGRPα mice, compared with knockout mice (Table 1). A summary of the two-way ANOVA interactions for analysis of periosteal bone formation is presented in Tables 2 and 3. A summary of load-induced endosteal bone formation in the CGRPα mice is given in S1 Text and S2 Text and S1 Fig. and S2 Fig. The relative contribution of Ps.MS/BS to endosteal bone formation is presented in S1 Table. A summary of the two-way ANOVA interactions for analysis of endosteal bone formation is presented in S2 Table and S3 Table.

**Table 1:** Relative contributions of mineralizing surface and mineral apposition rate to load-induced periosteal bone formation in CGRPα and CGRPβ wildtype and knockout mice.

	CGRPα	
	*Wildtype (%)*	*Knockout (%)*
**Ps.MS/BS**	10.6	2.0
**Ps.MAR**	26.7	62.3
**Ps.BFR/BS**	38.5	63.4
	**CGRPβ**	
**Ps.MS/BS**	6.0	10.2
**Ps.MAR**	12.2	−18.2
**Ps.BFR/BS**	21.1	−5.7

**Note**: Ps.MS/BS - periosteal mineralizing surface; Ps.MAR - periosteal mineral apposition rate; Ps.BFR/BS - periosteal bone formation rate. Data are derived from the mean values for the right ulna, which has loaded or sham loaded depending on group assignment and represent ((Right limb Load-Right limb Sham)-Right limb Sham)*100. CGRPα mice were bred on a C57BL/6 background. CGRPβ mice were bred on a Swiss background.

**Table 2:** Summary of two-way ANOVA results for load-induced periosteal bone formation in CGRPα and CGRPβ wildtype and knockout mice.

	CGRPα					
	*Wildtype*			*Knockout*		
	Ps.MS/BS	Ps.MAR	Ps.BFR/BS	Ps.MS/BS	Ps.MAR	Ps.BFR/BS
*Limb*	NS	*p*<0.001	*p*<0.001	NS *p* = 0.1	*p*<0.001	*p*<0.001
*Treatment*	*p*<0.005	NS *p* = 0.1	*p*<0.05	NS	NS *p* = 0.09	NS *p* = 0.1
*Interaction*	NS	NS	NS	*p*<0.05	*p*<0.001	*p*<0.005
	**CGRPβ**					
*Limb*	NS *p* = 0.07	*p*<0.005	*p*<0.005	NS	*p*<0.001	*p* = 0.001
*Treatment*	*p* = 0.13	NS	NS	NS *p = 0.06*	NS	NS
*Interaction*	NS	NS	NS	NS	NS *p* = 0.08	NS

**Note**: NS - not significant. *P* values<0.15 are also reported. Treatments were Sham, Load, or Block + Load.

**Table 3:** Summary of two-way ANOVA results for load-induced periosteal relative bone formation in CGRPα and CGRPβ wildtype and knockout mice.

	CGRPα		
	Ps.rMS/BS	Ps.rMAR	Ps.rBFR/BS
*Genotype*	NS *p* = 0.13	*p*<0.005	*p*<0.05
*Treatment*	*p*<0.05	NS	*p*<0.005
*Interaction*	*p*<0.05	*p* = 0.05	NS *p* = 0.10
	**CGRPβ**		
*Genotype*	NS	NS	NS
*Treatment*	NS	NS	NS
*Interaction*	NS	*p*<0.05	NS *p* = 0.06

**Note**: NS - not significant. *P* values<0.15 are also reported. Treatments were Sham, Load, or Block + Load.

### Load-induced bone formation responses are similar in CGRPβ wildtype and knockout mice

**Figure 5:**
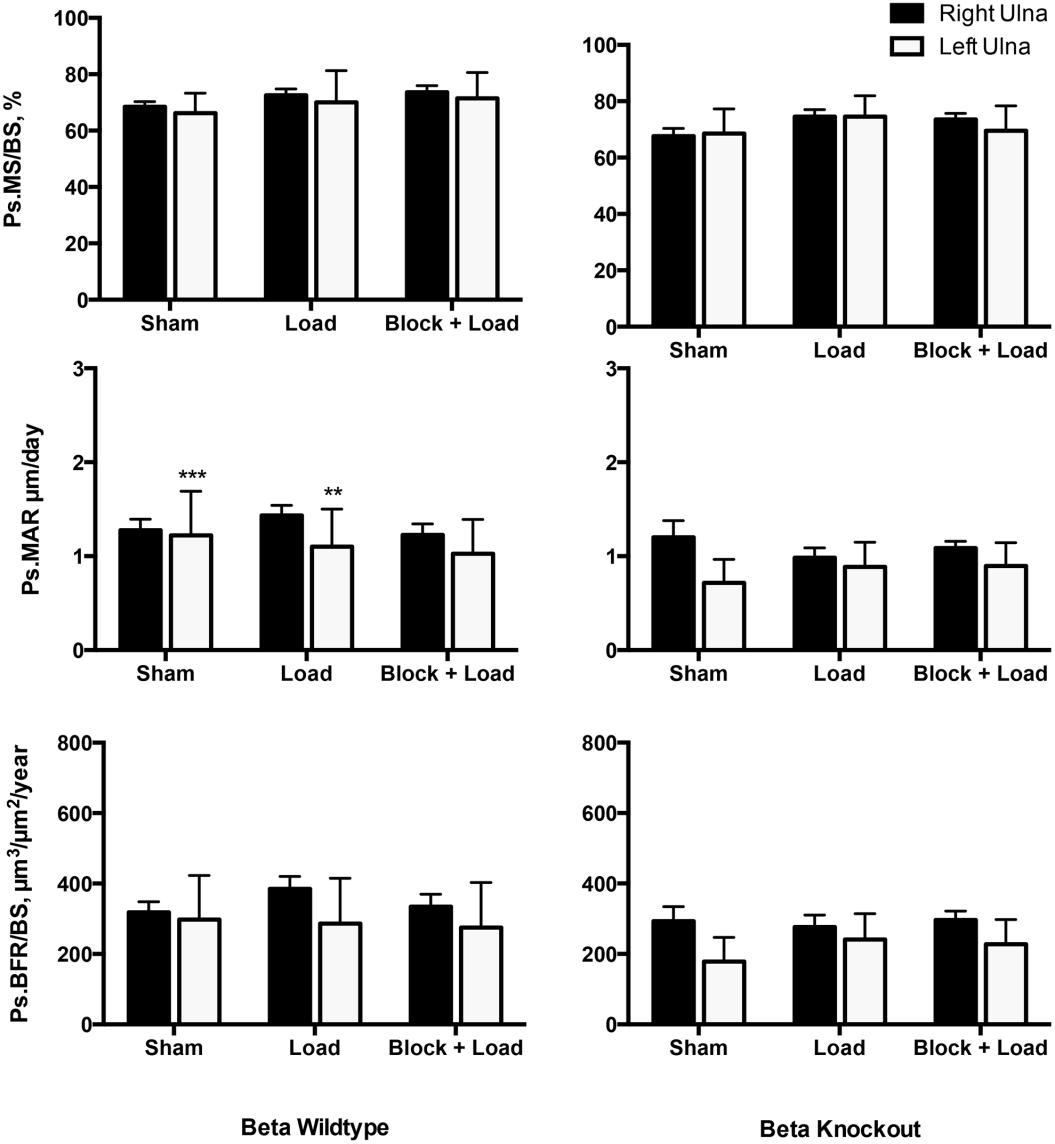
Load-induced periosteal bone formation responses are similar in CGRPβ wildtype and knockout mice. Overall, periosteal mineral apposition rate (Ps.MAR) in CGRPβ wildtype and knockout mice was significantly increased in the right ulna, when compared with the left ulna (*p*<0.005). Few other significant treatment effects were identified. **p<0.01; ***p<0.001 versus left ulna in associated CGRPβ knockout mice. Sham – sham loaded group, Load – loaded group, Block + Load – Brachial plexus anesthesia treatment before loading. n = 16–20 mice/group.

**Figure 6:**
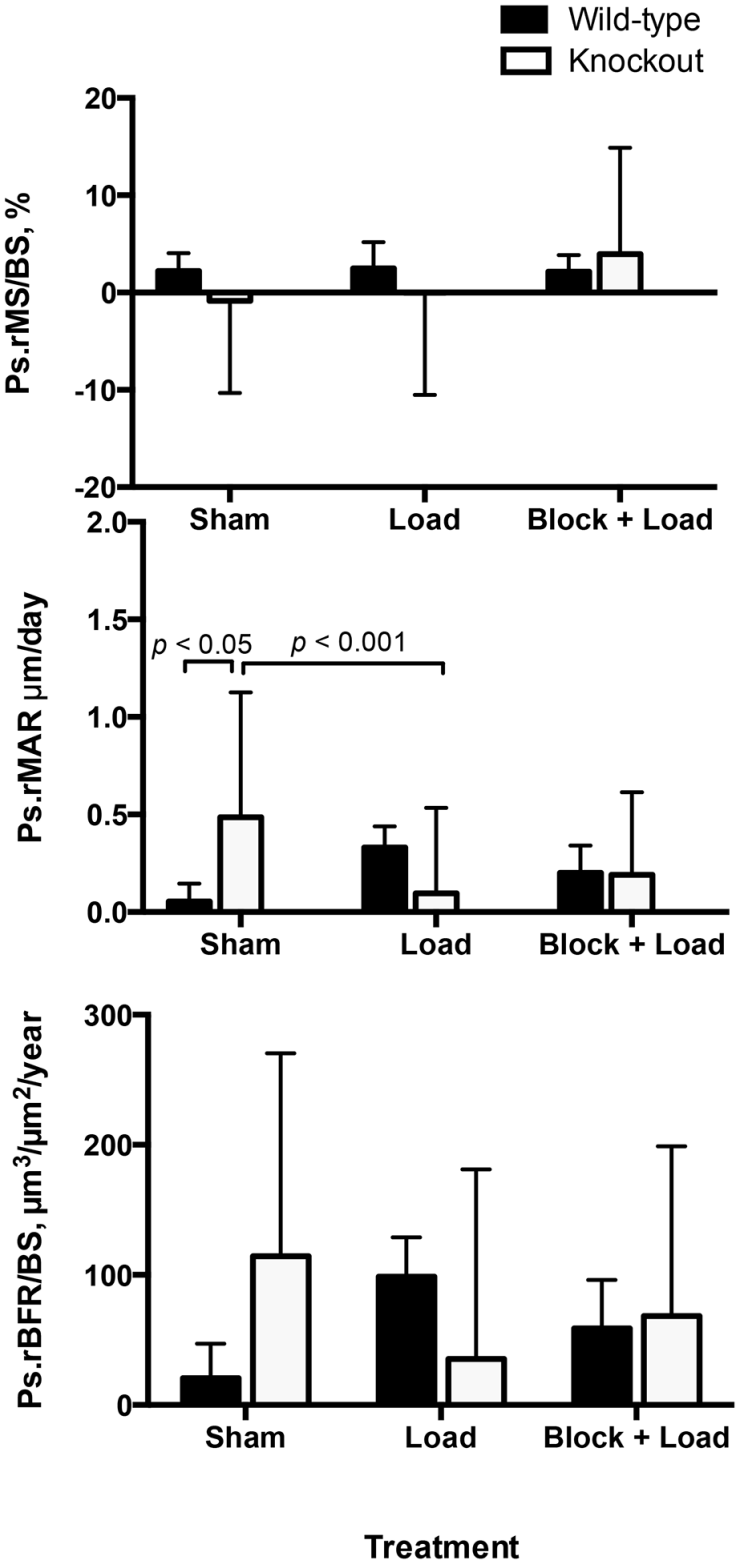
Adaptive periosteal bone responses in CGRPβ were not significantly influenced by mechanical loading treatments. In CGRPβ knockout mice, relative periosteal mineral apposition rate (Ps.rMAR) was increased in the Sham group compared with CGRPβ wildtype mice. Sham – sham loaded group, Load – loaded group, Block + Load – Brachial plexus anesthesia treatment before loading. n = 16–20 mice/group.

In contrast to the CGRPα mouse lines, few significant loading effects were found in CGRPβ wildtype and knockout mice on a Swiss background for both periosteal and endosteal bone formation. Ps.MS/BS in the loaded limb was not significantly influenced by the experimental treatments (*p*>0.05) (Fig. 5). Overall, Ps.MAR and Ps.BFR/BS were increased in the loaded limbs of CGRPβ wildtype (*p*<0.005) and knockout mice (*p*<0.005), relative to the contralateral left limb, although experimental treatment did not significantly influence Ps.MAR and Ps.BFR/BS (*p*>0.05) (Fig. 5). Ps.rMAR in CGRPβ knockout mice in the Sham group was significantly different from the Load group (*p*<0.001), and from the Sham-treated CGRPβ wildtype mice (*p*<0.05) (Fig. 6).

### Bone mineral density (BMD) is influenced by both CGRPα and CGRPβ signaling

**Figure 7:**
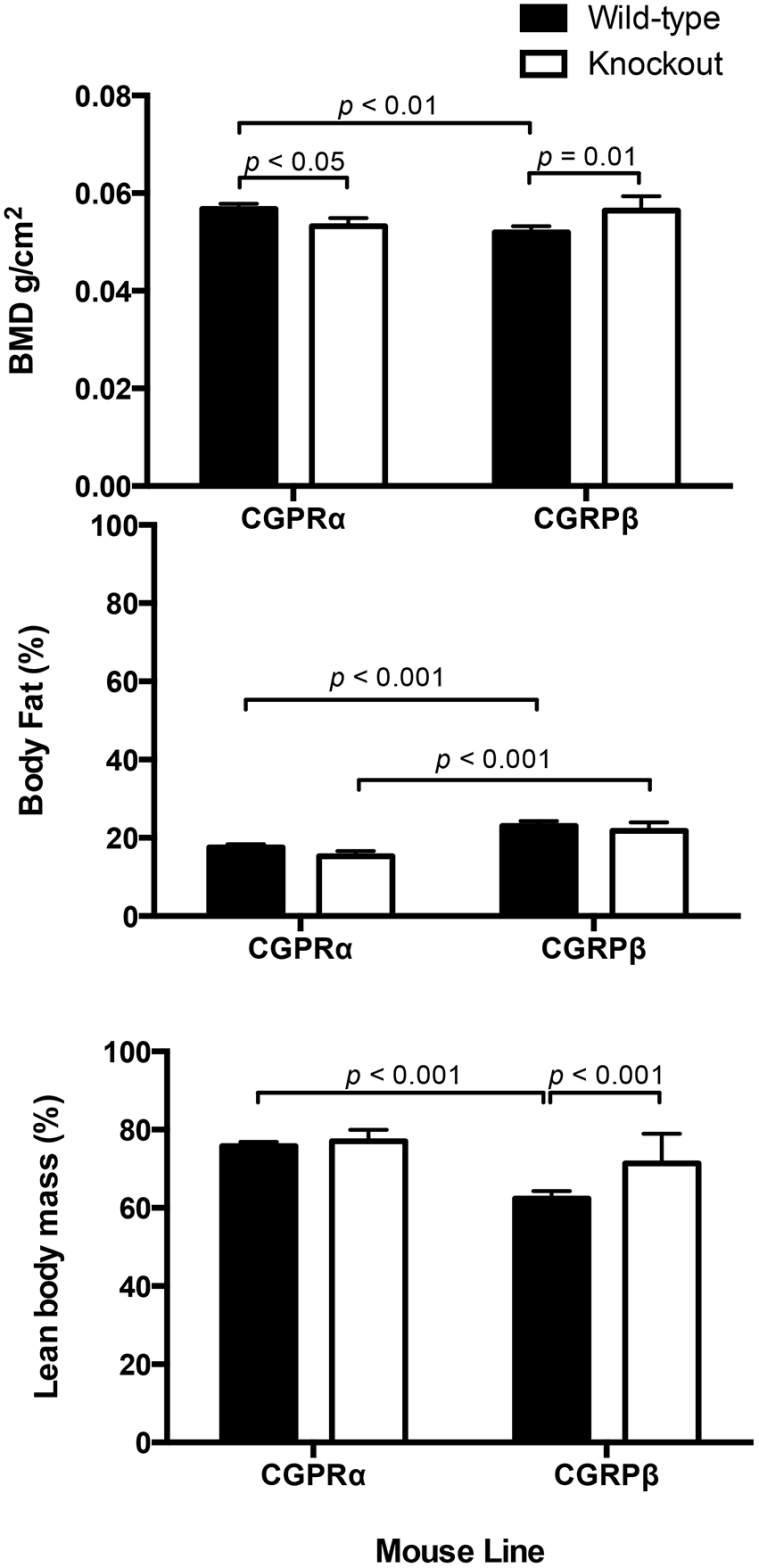
Deletion of the CGRP gene had opposite effects on bone mineral density (BMD) in CGRPα and CGRPβ mice. BMD was decreased in CGRPα knockout mice (*p*<0.05) and increased in CGRPβ knockout mice (*p* = 0.01), relative to their respective wildtype groups. In wildtype mice, BMD was decreased in Swiss background strain for the CGRPβ mice, relative to the C57BL/6 mice background strain for the CGRPα mice. C57BL/6 mice had a lower percentage body fat than Swiss mice (*p*<0001). In Swiss mice, deletion of CGRPβ function was associated with increased lean body mass (*p*<0.001). BMD was determined using a PIXImus densitometer, n = 5/group.

In CGRPα knockout mice, BMD was decreased relative to wildtype mice (*p*<0.05), whereas in CGRPβ knockout mice, BMD was increased relative to wildtype mice (*p* = 0.01) (Fig. 7). BMD was also increased in CGRPα wildtype mice, compared with CGRPβ wildtype mice (*p*<0.01) (Fig. 7).

### Body condition is influenced by mouse strain

Wildtype C57BL/6 mice were significantly leaner than wildtype Swiss mice (*p*<0.001) (Fig. 7). In Swiss mice, deletion of CGRPβ signaling was associated with increased lean body mass (*p*<0.001) (Fig. 7).

## Discussion

In this study, we examined the effects of CGRP signaling on bone responses to mechanical loading in wildtype and knockout mouse strains for both isoforms of CGRP, CGRPα and CGRPβ. Our goal was to investigate whether load-induced bone formation was neuronally regulated via CGRP signaling by studying ulnar responses to a short period of mechanical loading with and without neuronal blocking with BPA. Importantly, we found that CGRPα signaling had specific effects on periosteal mineralizing surface activation in response to mechanical loading. Deletion of CGRPα signaling reduced brachial plexus anesthesia blocking of Ps.MS/BS activation, but not Ps.MAR. Collectively, these results suggest that the CGRPα^+^ peptidergic innervation of bone regulates mineralizing surface activation in response to single short period of mechanical loading, and relate to the observation that bone CGRP concentrations are increased in response to cyclic ulna loading [25].

CGRPα has long been considered an osteoanabolic neuropeptide. Bone contains a dense meshwork of CGRP^+^ nerve fibers [13], with fiber sprouting occurring in a site-specific manner during fracture repair [21]. Furthermore, transgenic mice that overexpress CGRP have increased BMD [26]. In the present study, we found that CGRPα knockout mice have decreased BMD, reaffirming the results of earlier work [27]. In contrast, CGRPβ is not considered to have osteoanabolic effects based on in vitro studies [16]. We found CGRPβ knockout mice have increased BMD, suggesting that CGRPβ signaling actually has catabolic effects on skeletal metabolism *in* *vivo*.

In bone marrow stromal cell and osteoblast cell culture in vitro, CGRPα stimulates cellular differentiation and osteoblast proliferation [28], [29]. Activated CGRP receptors stimulate adenylyl-cyclase activity, upregulation of cAMP, and activation of protein kinase A (PKA) [30], [31]. It is widely accepted that canonical Wnt signaling is an important regulator of bone mass [32]. Canonical Wnt signaling relies on the cytosolic stabilization of β-catenin, a 130 amino acid protein that is phosphorylated by a glycogen synthase kinase (GSK-3β) in the absence of Wnt proteins [32]. CGRPα increases expression and concentrations of cytoplastic β-catenin and associated Wnt signaling by PKA-mediated inhibition of glycogen synthase kinase 3β (GSK-3β) [33]. In addition, CGRPα inhibits osteoblast apoptosis [33]. Collectively, these observations suggest that CGRP released from nerve fibers in bone exerts a local anabolic effect. Although many studies have examined osteoblast responses to fluid shear stress or other forms of mechanical loading in vitro, it has not been determined whether CGRP influences bone cell responses to mechanical loading in vitro.

Our in-vivo mouse studies suggest that CGRPα signaling has significant anabolic effects on osteoblast progenitor cell activation and proliferation leading to associated increases in Ps.MS/BS in response to mechanical loading. The principle source of CGRPα peptide in the bone microenviroment appears to be nerve fibers, since little secreted CGRP is released from bone cells [34], [35]. This concept could be directly addressed by experimental work examining local release in response to mechanical loading using immunohistochemistry, in-situ hydridization, or other related methods. Blocking of load-induced bone formation by BPA before mechanical loading suggests that the sensory nerve fibers in periosteum and bone are the source for local release of CGRPα [25] in response to bone loading. We also observed blocking of Ps.MS/BS in the contralateral left ulna of CGRPα wildtype mice suggesting that some degree of cross-talk between limbs occurs during mechanical loading [17], [18], [36]. Different to Ps.MS/BS, blocking of Ps.MAR in the right loaded ulna and blocking of Ps.rMAR was retained in CGRPα knockout mice, suggesting that once a periosteal bone-forming surface has been activated, CGRP signaling has little effect on osteoblast maintenance over time during functional adaptation to cyclic mechanical loading. However, retention of BPA-blocking on Ps.MAR in the CGRPα knockout mice suggests that neuronal signaling, presumably by a neurotransmitter other than CGRP, also significantly influences MAR. The relative contributions of MS/BS and MAR to periosteal bone formation were also altered with deletion of CGRPα signaling. The contribution of MS/BS activation to periosteal bone formation was much greater in CGRPα wildtype mice relative to CGRPα knockout mice. These observations suggest that mechanosensitivity to bone loading in the two C57BL/6 strains may not be identical. In contrast to the periosteal surface of the ulna, few significant loading effects were detected on the endosteal bone surface. This likely reflects the higher density of nerve fibers associated with the periosteal surface of long bones [37] and the lower peak strains present on bone surfaces closer to the neutral axis [38], [39].

Responses to mechanical loading of the ulna after deletion of CGRPβ signaling were different from that of CGRPα mice. Few significant treatment effects on periosteal and endosteal MS/BS and MAR were found. Overall, Ps.MAR was significantly higher in the right loaded ulna, when compared with the left contralateral ulna in both CGRPβ wildtype and knockout mice, suggesting a small adaptive response to mechanical loading was present in these groups of mice. Relative periosteal responses suggested that overall mechanosensitivity in Swiss mice was reduced compared with C57BL/6 mice. Adaptive responses to bone loading in mice are known to vary in different inbred strains of mice [40]. We found Ps.rMAR was increased in sham-loaded CGRPβ knockout mice, compared with sham-loaded CGRPβ wildtype mice and CGRPβ knockout mice in the Load treatment group. Taken together with the observation that CGRPβ wildtype mice have decreased BMD relative to CGRPβ knockout mice, these observations suggests that CGRPβ signaling does not contribute to the adaptive response to mechanical loading.

CGRPβ signaling has been little studied relative to CGRPα. The physiological effects of CGRP isoforms are thought to be similar, although tissue distribution is different [41]. In dorsal root ganglion, CGRPα mRNA is twice as abundant as CGRPβ mRNA, whereas CGRPβ mRNA is the only isoform expressed in the intestine in mice [42]. We found that deletion of CGRPβ signaling led to a gain in lean body mass. Although the biological explanation for this observation is unclear, it seems possible that this could be related to altered gut function. Current knowledge suggests that CGRPα is the predominant isoform for transmission of somatic pain sensations in peptidergic sensory fibers [42].

This study had several limitations. The fact that CGRPα and CGRPβ knockouts were bred on different background strains of mice limited interpretation of some of the results, because genetic background is known to influence mechanosensitivity in mice [40]. Further investigation of mechanosensitivity to loading in the four mouse strains studied may have aided interpretation of the results. Measurement of CGRP concentrations in bone [25] may have helped to strengthen our findings and aid interpretation of bone morphometry. Inclusion of a Sham+Block group would have provided specific information on whether short-term blockade of nerve conduction influences bone formation in the appendicular skeleton, although the present study was not designed to address this question. The brachial plexus blocking protocol used allows for temporary blockade of neuronal signals between the loaded limb and the central nervous system. Mice regained motor function in the right thoracic limb within 1 hour of recovery from general anesthesia. This is potentially advantageous over the nerve transection models that cause paralysis of the limb and permanent disuse that may confound any blocking effects on neuronal signaling [43]. A disadvantage is that large changes in bone volume are not expected, relative to rodent models in which multiple bouts of mechanical loading have been used [44]. Groups of animals with multiple periods of BPA before loading would have to be generated to extend the studies described in this report and other related work [17], [18].

In conclusion, significant mechanically-induced activation of periosteal mineralizing surface in mice was reduced by knockout of CGRPα signaling. In contrast, mineral apposition responses to mechanical loading were retained after CGRPα deletion. These results suggest that release of CGRP from sensory peptidgeric fibers in periosteum and bone has a functional role in bone responses to mechanical loading. Identifying, in detail, the pathway by which the peptidergic sensory innervation of bone detects and responds to mechanical loading events has the potential to reveal target molecules and signaling pathways for treatments that may enhance bone mass.

## Supporting Information

S1 Fig **Load-induced endosteal bone formation responses are different in CGRPα** **wildtype and knockout mice.** Overall, En.MS/BS in CGRPα knockout mice, but not wildtype mice was significantly increased in the right ulna, when compared with the left ulna (*p*<0.05). Endosteal mineral apposition rate (En.MAR) in the right loaded ulna of CGRPα knockout mice was increased in the Load group, relative to Sham (*p*<0.05). In CGRPα knockout mice, En.MAR was decreased in left contralateral ulna in the Block + Load group, relative to the Sham group (*p*<0.05). These differences were not found in wildtype mice. Sham – sham loaded group, Load – loaded group, Block + Load – BPA treatment before loading. n = 11–14 mice/group.
(DOCX)

S2 Fig **Load-induced endosteal bone formation responses are similar in CGRPβ** **wildtype and knockout mice.** In CGRPβ wildtype mice, endosteal mineralizing surface (Es.MS/BS) was increased in the right ulna in the Block + Load group compared with the contralateral ulna (*p*<0.05) and the right ulna in the Sham group (*p*<0.01). Sham – sham loaded group, Load – loaded group, Block + Load – BPA treatment before loading. n = 16–20 mice/group.
(DOCX)

S1 Table **Relative contributions of mineralizing surface and mineral apposition rate to load-induced endosteal bone formation in CGRPα and CGRPβ wildtype and knockout mice.**
(DOCX)

S2 Table **Summary of two-way ANOVA results for load-induced endosteal bone formation in CGRPα and CGRPβ wildtype and knockout mice.**
(DOCX)

S3 Table **Summary of two-way ANOVA results for load-induced endosteal relative bone formation in CGRPα and CGRPβ wildtype and knockout mice.**
(DOCX)

S1 Text **Load-induced endosteal bone formation responses in CGRPα wildtype and knockout mice.**
(DOCX)

S2 Text **Load-induced endosteal bone formation responses in CGRPβ wildtype and knockout mice.**
(DOCX)
