# Supplementary material for: Role of Calcitonin Gene-Related Peptide in Functional Adaptation of the Skeleton
Source: PLoS One. 2014 Dec 23;9(12):e113959. doi: 10.1371/journal.pone.0113959 (PMC4275203; doi:10.1371/journal.pone.0113959)

**Supporting Information**

To accompany Sample et al., PONE-D-14-02805

**Role of calcitonin gene-related peptide in functional adaptation of the skeleton**

**Figure S1. Load-induced endosteal bone formation responses are different in CGRPα** **wildtype and knockout mice**. Overall, En.MS/PS in CGRPα knockout mice, but not wild-type mice, was significantly increased in the right ulna when compared with the left ulna (*p* < 0.05). Endosteal mineral apposition rate (En.MAR) in the right loaded ulna of CGRPα knockout mice was increased in the Load group, relative to Sham (*p* < 0.05). In CGRPα knockout mice, En.MAR was decreased in left contralateral ulna in the Block + Load group, relative to the Sham group (*p* < 0.05). These differences were not found in wildtype mice. Sham – sham loaded group, Load - loaded group, Block + Load - BPA treatment before loading. n = 11-14 mice/group.


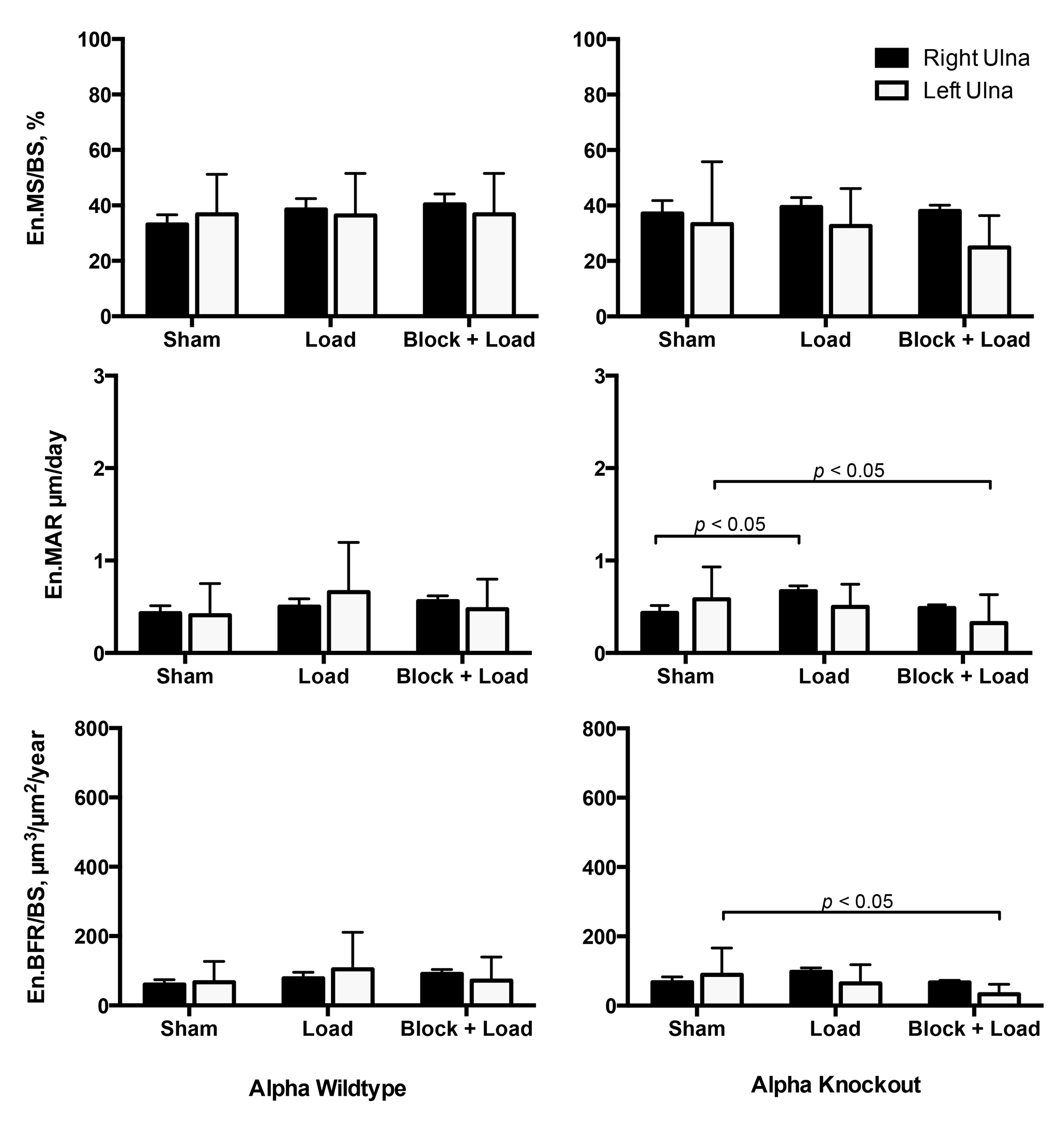

Supplement: S1 Fig — Load-induced endosteal bone formation responses are different in CGRPα wildtype and knockout mice. Overall, En.MS/BS in CGRPα knockout mice, but not wildtype mice was significantly increased in the right ulna, when compared with the left ulna (p<0.05). Endosteal mineral apposition rate (En.MAR) in the right loaded ulna of CGRPα knockout mice was increased in the Load group, relative to Sham (p<0.05). In CGRPα knockout mice, En.MAR was decreased in left contralateral ulna in the Block + Load group, relative to the Sham group (p<0.05). These differences were not found in wildtype mice. Sham – sham loaded group, Load – loaded group, Block + Load – BPA treatment before loading. n = 11–14 mice/group. (DOCX) [file pone.0113959.s001.docx]
