# Supplementary material for: Role of Calcitonin Gene-Related Peptide in Functional Adaptation of the Skeleton
Source: PLoS One. 2014 Dec 23;9(12):e113959. doi: 10.1371/journal.pone.0113959 (PMC4275203; doi:10.1371/journal.pone.0113959)

**Supporting Information**

To accompany Sample et al., PONE-D-14-02805

**Role of calcitonin gene-related peptide in functional adaptation of the skeleton**

**Figure S2.**  **Load-induced endosteal bone formation responses are similar in CGRPβ** **wildtype and knockout mice**. In CGRPβ wildtype, endosteal mineralizing surface (Es.MS/BS) was increased in the right ulna in the Block + Load group compared with the contralateral ulna (*p* < 0.05) and the right ulna in the Sham group (*p* < 0.01). Sham - sham loaded group, Load - loaded group, Block + Load - BPA treatment before loading. n = 16-20 mice/group.


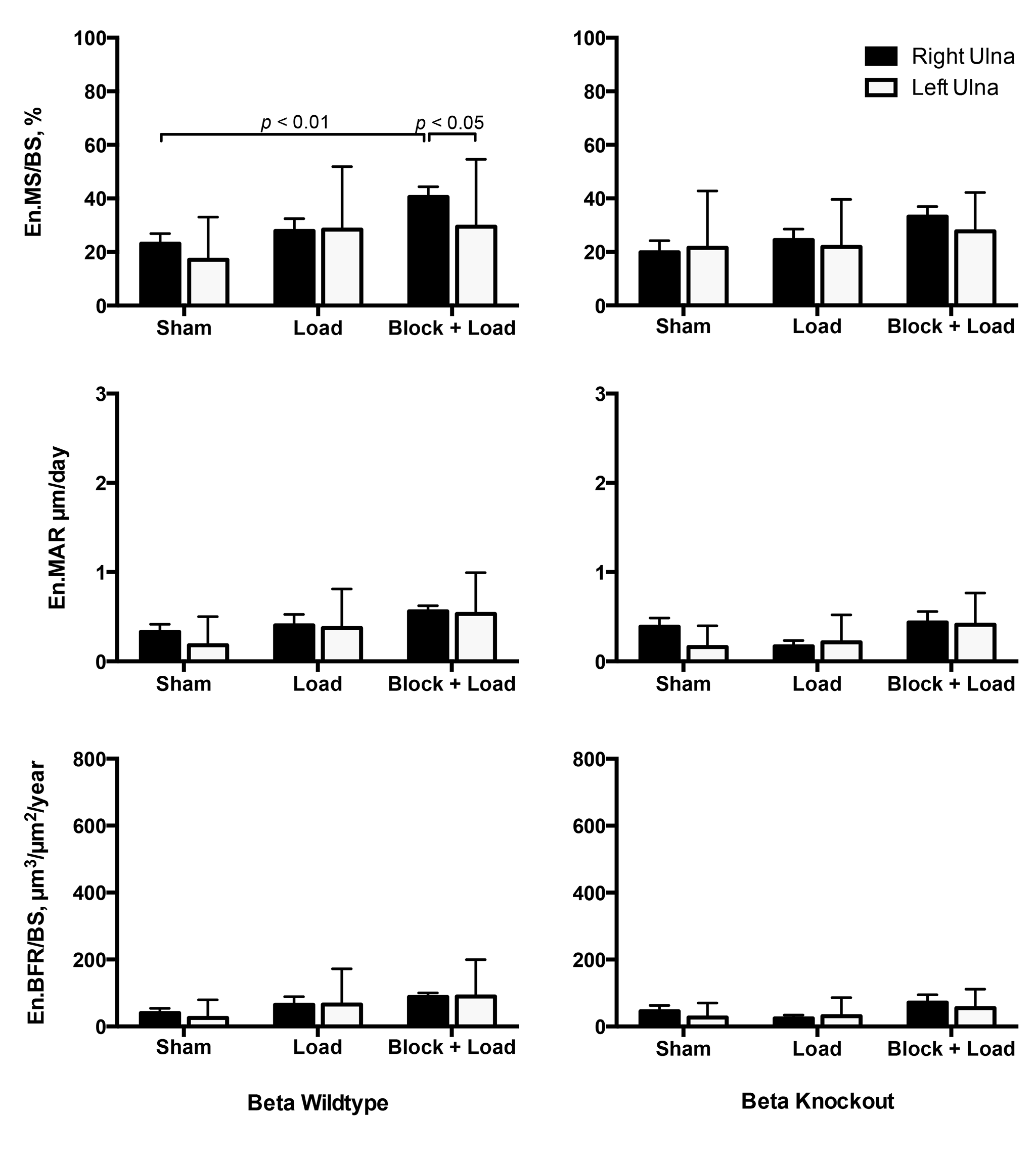

Supplement: S2 Fig — Load-induced endosteal bone formation responses are similar in CGRPβ wildtype and knockout mice. In CGRPβ wildtype mice, endosteal mineralizing surface (Es.MS/BS) was increased in the right ulna in the Block + Load group compared with the contralateral ulna (p<0.05) and the right ulna in the Sham group (p<0.01). Sham – sham loaded group, Load – loaded group, Block + Load – BPA treatment before loading. n = 16–20 mice/group. (DOCX) [file pone.0113959.s002.docx]
