# Supplementary material for: Role of Calcitonin Gene-Related Peptide in Functional Adaptation of the Skeleton
Source: PLoS One. 2014 Dec 23;9(12):e113959. doi: 10.1371/journal.pone.0113959 (PMC4275203; doi:10.1371/journal.pone.0113959)
Supplement: S1 Table — Relative contributions of mineralizing surface and mineral apposition rate to load-induced endosteal bone formation in CGRPα and CGRPβ wildtype and knockout mice. (DOCX) [file pone.0113959.s003.docx]

**Table S1. Relative contributions of mineralizing surface and mineral apposition rate to load-induced endosteal bone formation in CGRPα and CGRPβ wildtype and knockout mice**

|  | **CGRPα** | |
| --- | --- | --- |
|  | *Wildtype (%)* | *Knockout (%)* |
| **En.MS/BS** | 16.4 | 6.3 |
| **En.MAR** | 16.2 | 54.1 |
| **En.BFR/BS** | 30.1 | 43.6 |
|  | **CGRPβ** | |
| **En.MS/BS** | 20.7 | 23.4 |
| **En.MAR** | 21.9 | -56.5 |
| **En.BFR/BS** | 60.5 | -47.4 |

**Note**: En.MS/BS - endosteal mineralizing surface; En.MAR - endosteal mineral apposition rate; En.BFR - endosteal bone formation rate. Data are derived from the right ulna, which has loaded or sham loaded depending on group assignment and represent ((Right limb Load-Right limb Sham)/Right limb Sham)*100. CGRPα were bred on a C57BL/6 background. CGRPβ were bred on a Swiss background.
