# Supplementary material for: Role of Calcitonin Gene-Related Peptide in Functional Adaptation of the Skeleton
Source: PLoS One. 2014 Dec 23;9(12):e113959. doi: 10.1371/journal.pone.0113959 (PMC4275203; doi:10.1371/journal.pone.0113959)
Supplement: S2 Table — Summary of two-way ANOVA results for load-induced endosteal bone formation in CGRPα and CGRPβ wildtype and knockout mice. (DOCX) [file pone.0113959.s004.docx]

**Table S2. Summary of two-way ANOVA results for load-induced endosteal bone formation in CGRPα and CGRPβ wildtype and knockout mice**

|  | **CGRPα** | | | | | |
| --- | --- | --- | --- | --- | --- | --- |
|  | *Wildtype* | | | *Knockout* | | |
|  | **En.MS/BS** | **En.MAR** | **En.BFR** | **En.MS/BS** | **En.MAR** | **En.BFR** |
| *Limb* | NS | NS | NS | NS | NS | NS |
| *Treatment* | NS | NS | NS | *p* < 0.05 | *p* < 0.05 | NS |
| *Interaction* | NS | NS | NS | NS | NS | NS |
|  | **CGRPβ** | | | | | |
| *Limb* | NS | NS | NS | NS | NS | NS |
| *Treatment* | *p* < 0.05 | NS | NS | NS | NS | NS |
| *Interaction* | *p* < 0.05 | NS | NS | NS | NS | NS |

**Note**: NS - not significant. Treatments were Sham, Load, or Block + Load.
