# Supplementary material for: Role of Calcitonin Gene-Related Peptide in Functional Adaptation of the Skeleton
Source: PLoS One. 2014 Dec 23;9(12):e113959. doi: 10.1371/journal.pone.0113959 (PMC4275203; doi:10.1371/journal.pone.0113959)
Supplement: S3 Table — Summary of two-way ANOVA results for load-induced endosteal relative bone formation in CGRPα and CGRPβ wildtype and knockout mice. (DOCX) [file pone.0113959.s005.docx]

**Table S3. Summary of two-way ANOVA results for load-induced endosteal relative bone formation in CGRPα and CGRPβ wildtype and knockout mice**

|  | **CGRPα** | | |
| --- | --- | --- | --- |
|  | **En.rMS/BS** | **En.rMAR** | **En.rBFR** |
| *Genotype* | NS | NS | NS |
| *Treatment* | NS | NS | NS |
| *Interaction* | NS | NS | NS |
|  | **CGRPβ** | | |
| *Genotype* | NS | NS | NS |
| *Treatment* | NS | NS | NS |
| *Interaction* | NS | NS | NS |

**Note**: NS – not significant.
