# Supplementary material for: Role of Calcitonin Gene-Related Peptide in Functional Adaptation of the Skeleton
Source: PLoS One. 2014 Dec 23;9(12):e113959. doi: 10.1371/journal.pone.0113959 (PMC4275203; doi:10.1371/journal.pone.0113959)
Supplement: S1 Text — Load-induced endosteal bone formation responses in CGRPα wildtype and knockout mice. (DOCX) [file pone.0113959.s006.docx]

**Supporting Information**

To accompany Sample et al., PONE-D-14-02805

**Role of calcitonin gene-related peptide in functional adaptation of the skeleton**

**Text S1**

**Load-induced endosteal bone formation responses in CGRPα wildtype and knockout mice**

Few significant changes in endosteal bone formation were detected in response to bone loading in CGRPα wildtype and knockout groups of mice (**Fig. S1**). No significant effects on En.MS/BS were found in CGRPα wildtype mice. In CGRPα knockout mice, endosteal bone formation overall in the loaded right limb was increased relative to the left contralateral limb (*p* < 0.05) (**Fig. S1**). En.MAR in the loaded ulna was increased in CGRPα knockout mice relative to sham (*p* < 0.05). En.MAR and En.BFR/BS in the contralateral ulna were decreased in the Block+Load group in CGRPα knockout mice, relative to Sham (*p* < 0.05) (**Fig. S1**).
