# Supplementary material for: Role of Calcitonin Gene-Related Peptide in Functional Adaptation of the Skeleton
Source: PLoS One. 2014 Dec 23;9(12):e113959. doi: 10.1371/journal.pone.0113959 (PMC4275203; doi:10.1371/journal.pone.0113959)
Supplement: S2 Text — Load-induced endosteal bone formation responses in CGRPβ wildtype and knockout mice. (DOCX) [file pone.0113959.s007.docx]

**Supporting Information**

To accompany Sample et al., PONE-D-14-02805

**Role of calcitonin gene-related peptide in functional adaptation of the skeleton**

**Text S2**

**Load-induced endosteal bone formation responses in CGRPβ mice**

Few significant changes in endosteal bone formation were detected in response to bone loading in CGRPβ wildtype and knockout groups of mice (**Fig. S2**). In the right Block+Load ulna, En.MS/BS was higher relative to Sham (*p* < 0.01) and Loaded groups (*p* = 0.056) in CGRPβ wildtype mice (**Fig. S2**). En.MAR was not significantly influenced by treatment or mouse type.
